# Reverse Total Shoulder Arthroplasty for Failed Osteosynthesis in Proximal Humerus Fractures: Outcomes and Challenges

**DOI:** 10.7759/cureus.101390

**Published:** 2026-01-12

**Authors:** Luís B Pinto, João Alves, Eduardo Ferreira, Herculano Nascimento, António Miranda, Tiago P Torres

**Affiliations:** 1 Orthopaedics, Unidade Local de Saúde de Viseu Dão-Lafões, Viseu, PRT; 2 Orthopaedics, Unidade Local de Saúde Entre Douro e Vouga, Santa Maria da Feira, PRT

**Keywords:** avascular necrosis, failed osteosynthesis, proximal humerus fractures, reverse total shoulder arthroplasty, salvage procedure

## Abstract

Background

Proximal humerus fractures (PHF) are among the most common humeral fractures, particularly affecting elderly individuals, often following low-energy trauma. While conservative treatment is often preferred in geriatric patients, surgical management remains indicated for displaced fractures. Reverse total shoulder arthroplasty (RTSA) has increasingly been used as a primary treatment option for complex PHF, especially Neer three and four-part patterns. However, in the setting of failed osteosynthesis, there is a lack of evidence regarding the role and outcomes of RTSA.

Methods

A retrospective study of nine patients who underwent RTSA for failed osteosynthesis of PHF between 2018 and 2023 was conducted. Demographic and clinical data were collected from electronic medical records. The patients were clinically reassessed, and preoperative imaging was reviewed to identify failure mechanisms. Functional outcomes were assessed using the Constant Score, Visual Analog Scale (VAS) for pain, and active range of motion (AROM), with a minimum follow-up of 12 months.

Results

The cohort included predominantly female patients (mean age: 67 years). Most initial fractures were Neer two-part (n=6; 67%), treated with open reduction and internal fixation (ORIF) using plates and screws (n=5; 83%) or a proximal humeral nail (n=1; 17%). Two Neer three-part fractures (n=2; 22%) and one metaphyseal fracture (n=1; 11%) were also included. Failure mechanisms included varus collapse with screw pull-out (n=4; 45%), nail pull-out (n=2; 22%), avascular necrosis (n=2; 22%) and one case of non-union (11%). All patients presented with persistent pain and dysfunction as indications for RTSA. A standardized surgical technique was employed in all cases and tuberosity reattachment was attempted when feasible. At the one-year follow-up, mean AROM was 90° of abduction, 110° of forward flexion, external rotation reaching the nape of the neck, and internal rotation reaching the lateral side of the buttock. Mean Constant Score improved from 35 preoperatively to 88 postoperatively, and VAS scores decreased from seven to three. One case of postoperative traumatic dislocation was recorded. Mean follow-up duration was 27 months.

Conclusion

RTSA is an effective salvage procedure following failed osteosynthesis of PHF, offering reliable pain relief and functional improvement. Despite a small sample size, this study reinforces the utility of RTSA in complex revision scenarios. Calcar comminution and poor bone quality were identified as potential risk factors for initial fixation failure, emphasizing the importance of anatomic reduction and metaphyseal screw placement during primary osteosynthesis.

## Introduction

Proximal humerus fractures (PHFs) are the most common fractures of the humerus. They account for 4% to 10% of all fractures and approximately half of all upper limb fractures. PHF occur more frequently in elderly women and are generally associated with low-energy trauma, making them the third most common osteoporotic fractures [1].

The management of these fractures in the geriatric population is predominantly conservative [2]. However, in younger patients and in cases of complex fracture patterns (e.g., Neer three- and four-part fractures [3]) with significant displacement, surgical treatment is indicated unless medical risk factors deem the patient unfit for surgery. Surgical options comprise open reduction and internal fixation (ORIF) or closed reduction and internal fixation (CRIF), as well as reverse total shoulder arthroplasty (RTSA) [1,4,5]. In recent years, RTSA has been increasingly used as the primary surgical treatment for PHFs in Neer three- and four-part fractures in active elderly patients, providing more predictable outcomes compared to osteosynthesis and hemiarthroplasty [6-8]. This trend is due to the complications often associated with ORIF or CRIF, such as screw cut-out, malunion of the tuberosities, glenoid erosion, and avascular necrosis of the humeral head [6-8].

There is still controversy regarding the minimum age for indicating primary RTSA in PHFs. However, in cases of osteosynthesis failure, the options typically include either revision surgery or conversion to RTSA [7-11]. Currently, there is a lack of robust scientific evidence regarding the role and outcomes of RTSA in the treatment of osteosynthesis failures and proximal humerus non-union [10,12-16].

The aim of this study is to describe nine cases of osteosynthesis failure and non-union after osteosynthesis in PHFs treated with RTSA. The authors sought to analyze the clinical and functional outcomes of RTSA following failed osteosynthesis, as well as the causes and potential risk factors for osteosynthesis failure as a secondary objective.

## Materials and methods

This is a retrospective observational study, which included patients who underwent revision surgery with RTSA following the failure of proximal humerus osteosynthesis. The study was conducted at a single tertiary care institution. Cases treated between January 2018 and December 2023 with a minimum follow-up of 12 months were eligible for inclusion.

Failure of osteosynthesis was defined as the presence of complications such as non-union, fixation failure, screw cut-out, implant loosening or humeral head avascular necrosis, requiring revision surgery. A total of nine patients with failed proximal humerus osteosynthesis, who were subsequently treated with RTSA, were included.

Patients’ electronic medical records were retrospectively reviewed to collect demographic data, comorbidities, fracture characteristics, type of initial osteosynthesis, and causes for fixation failure. All patients were also clinically reassessed at the time of data collection to obtain updated clinical and functional outcomes.

The primary objective was to evaluate the clinical and functional outcomes of RTSA as a salvage procedure for proximal humerus osteosynthesis failure. A secondary objective was to identify potential risk factors associated with osteosynthesis failure. Clinical assessment included pain evaluation and shoulder function using standardized outcome measures with the Visual Analog Scale (VAS) and Constant-Murley score [17-19]. In all the procedures, the surgical approach, implant selection, and postoperative rehabilitation protocols were standardized according to the institutional practice.

According to the institutional policy, formal approval from an ethics committee was not required for retrospective observational studies based on anonymized clinical data. Therefore, ethics approval and informed consent were waived for this study.

## Results

There was a predominance of female patients (n=8; 89%), with a mean age of 67 years at the time of RTSA (Table 1).

**Table 1:** Patient demographics RTSA: reverse total shoulder arthroplasty

Total patients (n)	9
Male patients	1
Female patients	8
Mean age at RTSA (range in years)	67 (54-75)
Left side	3
Right side	6
Mean follow-up (months)	22 ± 4

In six cases (67%), the initial fracture was classified as a Neer two-part fracture, five (83%) of which were treated with ORIF using plates and screws, and one case (17%) was treated with a proximal humeral nail (Stryker®, Michigan, USA). Two cases (22%) involved Neer three-part fractures, both treated with ORIF using plates and screws (Stryker®, Michigan, USA). The remaining case (11%) was a proximal humeral metaphyseal fracture treated with a proximal humeral nail (Table 2).

**Table 2:** Cases with fracture classification and chosen fixation method ORIF: open reduction and internal fixation; CRIF: closed reduction and internal fixation

Case	Patient	Fracture (Neer classification)	Surgery
1	Female, 75y	2-part	ORIF w/ plate and screws
2	Female, 73y	2-part	ORIF w/ plate and screws
3	Female, 54y	2-part	ORIF w/ plate and screws
4	Female, 70y	2-part	CRIF w/ proximal humeral nail
5	Female, 68y	2-part	ORIF w/ plate and screws
6	Female, 53y	3-part	ORIF w/ plate and screws
7	Female, 70y	2-part	ORIF w/ plate and screws
8	Male, 65y	3-part	ORIF w/ plate and screws
9	Female, 71y	Proximal humeral metaphysis	CRIF w/ proximal humeral nail

Causes of failure are summarized in Table 3.

**Table 3:** Causes of failure

Case	Patient	Fracture (Neer classification)	Cause of failure
1	Female, 75y	2-part	Varus collapse
2	Female, 73y	2-part	Non-union
3	Female, 54y	2-part	Varus collapse
4	Female, 70y	2-part	Nail pull-out
5	Female, 68y	2-part	Varus collapse
6	Female, 53y	3-part	Avascular necrosis
7	Female, 70y	2-part	Varus collapse
8	Male, 65y	3-part	Avascular necrosis
9	Female, 71y	Proximal humeral metaphysis	Nail pull-out

**Figure 1:**
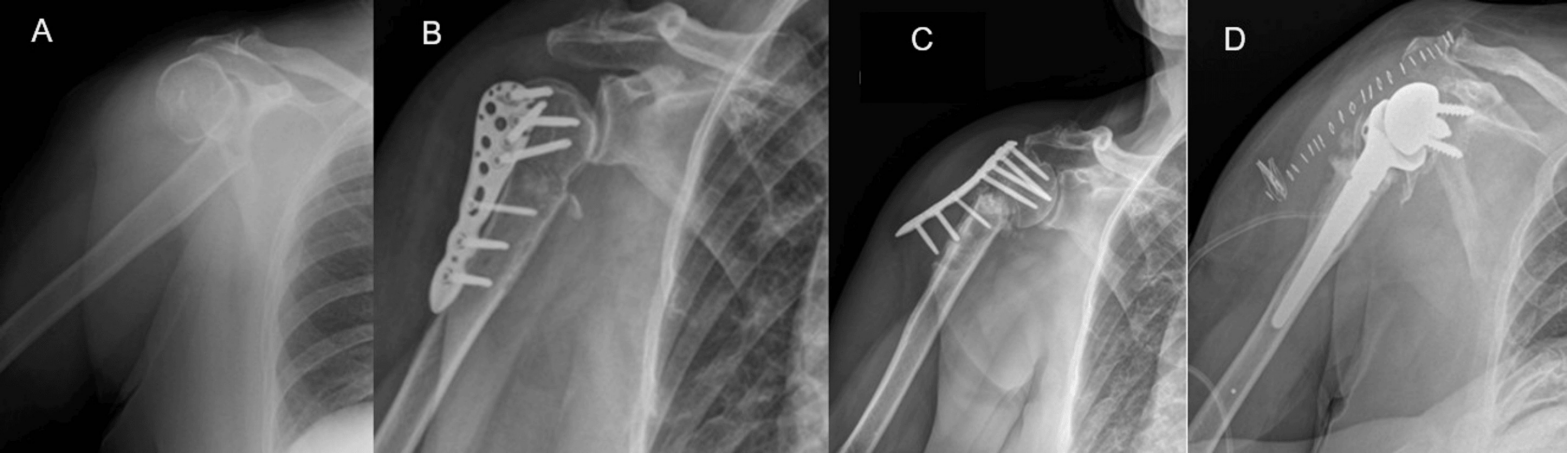
Case 1 A) Two-part PHF B) ORIF with plate and screws C) Varus collapse with screw pull-out D) RTSA PHF: proximal humerus fracture; ORIF: open reduction and internal fixation; RTSA: reverse total shoulder arthroplasty

**Figure 2:**
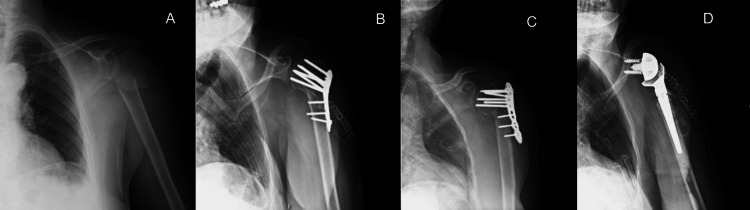
Case 3 A) Two-part PHF B) ORIF with plate and screws C) Varus collapse with screw pull-out D) RTSA PHF: proximal humerus fracture; ORIF: open reduction and internal fixation; RTSA: reverse total shoulder arthroplasty

**Figure 3:**
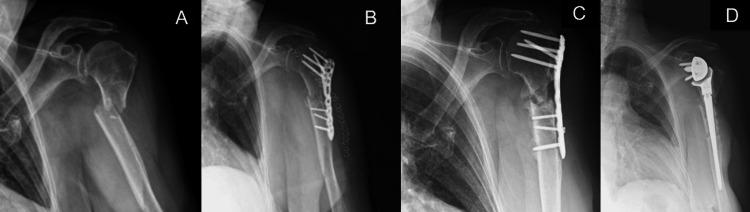
Case 2 A) Two-part PHF B) ORIF with plate and screws C) At 9-month follow-up, Non-union D) RTSA PHF: proximal humerus fracture; ORIF: open reduction and internal fixation; RTSA: reverse total shoulder arthroplasty

**Figure 4:**
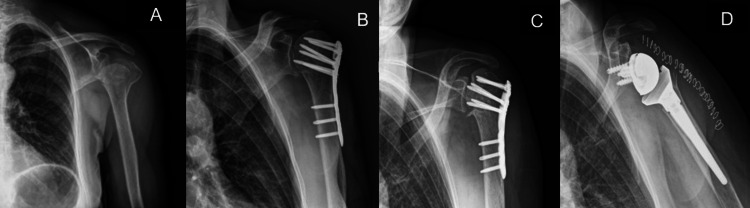
Case 6 A) Three-part PHF B) ORIF with plate and screws C) Avascular necrosis D) RTSA PHF: proximal humerus fracture; ORIF: open reduction and internal fixation; RTSA: reverse total shoulder arthroplasty

**Figure 5:**
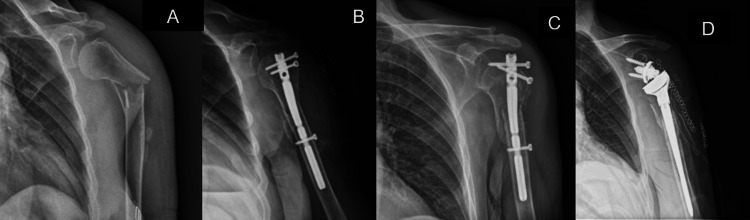
Case 9 A) Proximal humeral metaphysis fracture B) CRIF with proximal humeral nail C) Proximal nail pull-out D) RTSA CRIF:  closed reduction and internal fixation; RTSA: reverse total shoulder arthroplasty

In all cases, the indication for RTSA was persistent pain and significant shoulder dysfunction. Figures 1-5 provide a clinical and radiological description of some of the cases.

In all cases, a preoperative infection screening was performed through laboratory tests, and a preoperative CT scan was conducted for surgical planning. Additionally, four intraoperative samples were collected for cytobacteriological examination in all patients, with all results being negative for infection. The removal of osteosynthesis material was performed in the same surgical procedure as the RTSA, except for one case in which RTSA was carried out nine months after hardware removal.

The surgical technique used was the same for all patients, and all procedures were performed by experienced shoulder surgeons. Patients were positioned in a "beach chair" position under general anesthesia with an interscalene block. The surgical approach used was the deltopectoral approach, and a tenodesis of the long head of the biceps tendon was performed in cases where it was still present.

**Figure 6:**
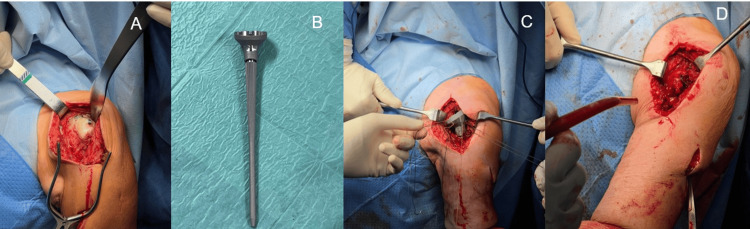
Case 9 A) Proximal humeral nail pull-out with rotator cuff lesion B) Long humeral stem (LIMA®) C) and D) Reinsertion of tuberosities with the technique describe by Boileau et al. [20]. Implant details: LIMA® (LimaCorporate S.p.A., San Daniele del Friuli, Udine, Italy)

RTSA was performed using a LIMA® implant (LimaCorporate S.p.A., San Daniele del Friuli, Udine, Italy) in all patients. Whenever possible, tuberosity preservation and reattachment were attempted, provided that the bone stock was deemed satisfactory based on the surgeon’s assessment and following the technique described by Boileau et al. [20-24]. Reinsertion of the tuberosities was achieved in four patients (44%) (Figure 6).

In two cases (22%), a cemented humeral stem was used, with one of them requiring a long stem. The decision to cement the humeral stem was made intraoperatively. The average length of hospital stay was three days, with all patients receiving perioperative antibiotic prophylaxis with cefazolin for 24 hours. Rehabilitation was initiated during hospitalization, with an average total rehabilitation period of one year and six months.

**Figure 7:**
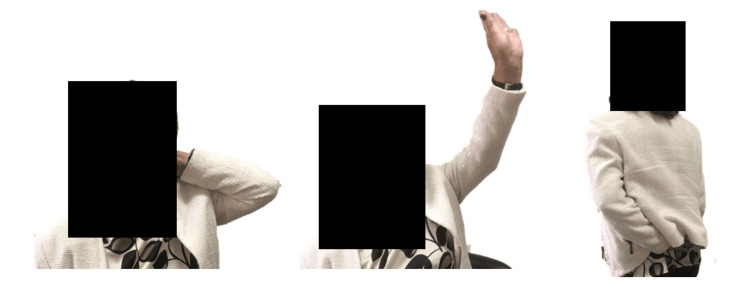
Case 9 Patient AROM six months after RTSA; AROM: active range of motion; RTSA: reverse total shoulder arthroplasty

At the one-year follow-up, the mean active range of motion (AROM) was 90° of abduction, 110° of forward flexion, external rotation reaching the nape of the neck on average, and internal rotation reaching the lateral side of the buttock (Table 4 and Figure 7).

**Table 4:** Clinical and functional outcomes

AROM	Mean value
Abduction	80º
Forward elevation	85º
External rotation	Nape
Internal rotation	Lateral buttock
Pain Visual Analog Scale [17]	
Preoperative	7
Postoperative	3
Constant Score [18,19]	
Preoperative	65
Postoperative	88

Regarding complications after RTSA, only one case (11%) of traumatic dislocation was reported, which occurred three months postoperatively following a fall. The mean pre-RTSA VAS score was seven, decreasing to three after RTSA. The mean pre-RTSA Constant-Murley Score was 35, improving to 88 postoperatively, with the lowest post-RTSA Constant-Murley Score observed in the case of traumatic dislocation. The average time between the initial surgery and conversion to RTSA was 11.5 months, and the mean follow-up period after RTSA was 27 months.

## Discussion

Performing RTSA after the failure of osteosynthesis in a PHF requires careful planning and execution by experienced shoulder surgeons. The presence of bone defects, poor residual bone stock quality, rotator cuff lesions and the condition of the surrounding soft tissues are key concerns in these cases [25-30]. Additionally, ruling out infection as a cause of osteosynthesis failure is mandatory [25,27-28].

RTSA is currently recognized in the literature as an excellent option as a revision procedure for cases of failed osteosynthesis in PHFs [28]. A recent study by Caldaria et al. demonstrated that RTSA, as a salvage procedure in such situations, provides satisfactory and durable outcomes compared to alternative therapeutic options, which have shown limited success [10,31,32]. 

In a study by Shannon et al., RTSA performed as a salvage procedure after unsuccessful PHF osteosynthesis exhibited a slightly higher complication rate compared to primary RTSA, but the revision and reoperation rate, as well as clinical outcomes and shoulder function, were comparable [33]. In another study, Sebastia-Forcada et al. compared the outcomes of primary RTSA versus secondary arthroplasty after failed proximal humeral locking plate fixation and demonstrated that patients who were revised to RTSA secondarily had slightly lower functional scores and higher complication rates compared with patients treated with primary RTSA but had significant functional improvement and pain relief compared with their preoperative status [16].

In our case series, RTSA resulted in significant improvements in both pain and mobility, with a mean post-RTSA Constant-Murley score of 88. Current literature reports high complication rates for RTSA following failed ORIF in PHF, highlighting dislocation as the most common complication [13]. We observed only one case (11%) of traumatic dislocation in our series, while less frequently, aseptic loosening, infection and periprosthetic fracture are also concerning complications. Boileau et al. suggested that proximal humeral bone loss could lead to an increased rate of aseptic loosening of the humeral component in RTSA [21-23,34]. Although no cases of aseptic loosening were recorded in our series, the follow-up period remained relatively short.

Lauren et al. reported a 20% reoperation rate in patients undergoing RTSA for proximal humerus non-union [13]. This high reoperation rate was not observed in our study, possibly due to the small sample size. Notably, in four out of the six cases (67%) of Neer two-part fractures treated with plates and screws (patients one, three, five and seven), osteosynthesis failure resulted from intraoperative malreduction, leading to varus collapse and screw pull-out, highlighting the importance of proper reduction during ORIF. Additionally, in all four of these cases, significant calcar comminution was present, which may have contributed to the osteosynthesis failure. Furthermore, in two cases (22%) (patients four and nine) of the proximal humeral nail pull-out, the authors retrospectively considered that, given the patients’ age (>70 years), they likely had poor, osteoporotic bone stock unsuitable for osteosynthesis and would have been better treated with primary RTSA. In the two cases (22%) with avascular necrosis (patients six and eight), both had preoperative Herthel Criteria (disrupted medial hinge as well as a displaced anatomical neck fracture, with >45º of angulation) with an increased risk of humeral head ischemia. It is also important to mention that the only case of non-union of our series (patient two) occurred in a female patient who was a smoker and had osteoporosis.

Our study has some limitations, including its retrospective nature, small sample size, absence of a comparison group (e.g., revision ORIF or primary RTSA), potential selection bias, heterogeneity in fracture patterns and failure mechanisms, which limits generalizability and precludes robust subgroup analysis and relatively short follow-up period.

## Conclusions

Our case series adds to the growing body of evidence supporting RTSA as a complex but effective salvage surgical treatment for failed osteosynthesis of PHFs, achieving good clinical and functional outcomes. Nevertheless, the results are not always predictable, and the complication rate remains considerable. Additionally, observations from our retrospective case series suggest that certain characteristics of the initial fracture pattern, such as calcar comminution and poor bone quality, may be associated with an increased risk of osteosynthesis failure. Given the retrospective nature of our study as well as our small sample size, these findings should be interpreted in the context of the existing literature rather than as definitive risk factors. Nonetheless, they reinforce previously reported principles regarding the importance of meticulous surgical technique, including anatomical restoration of the calcar and adequate metaphyseal support, to reduce the risk of varus collapse in surgical neck fractures treated with locking plates.
